# Supplementary figures and images for: Non-cognate immunity proteins provide broader defenses against interbacterial effectors in microbial communities
Source: eLife. 2025 Sep 30;12:RP90607. doi: 10.7554/eLife.90607 (PMC12483513; doi:10.7554/eLife.90607)

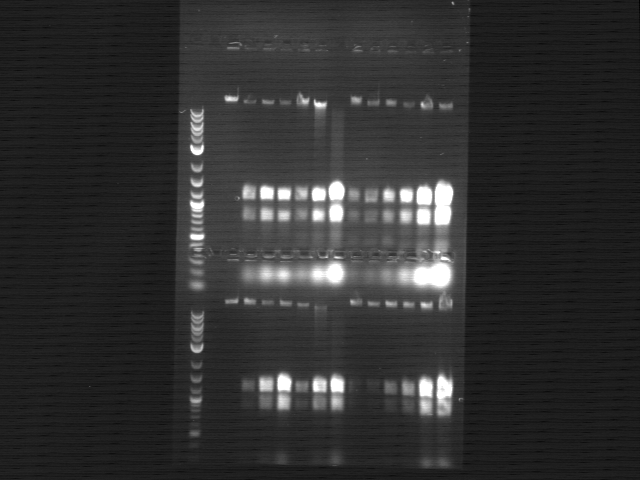

Supplement: Figure 1—source data 2. [file elife-90607-fig1-data2.zip › Figure 1-source data 2/Figure 1C-top and bottom_2018-08-29 15hr 50min.tif]

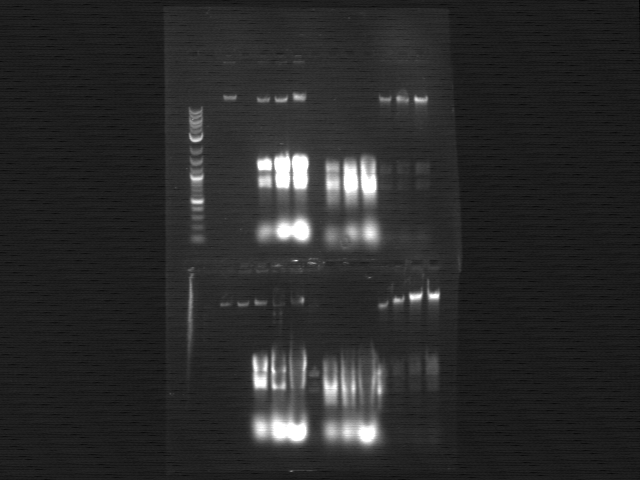

Supplement: Figure 1—source data 2. [file elife-90607-fig1-data2.zip › Figure 1-source data 2/Figure 1E-top_2018-08-22 14hr 57min.tif]

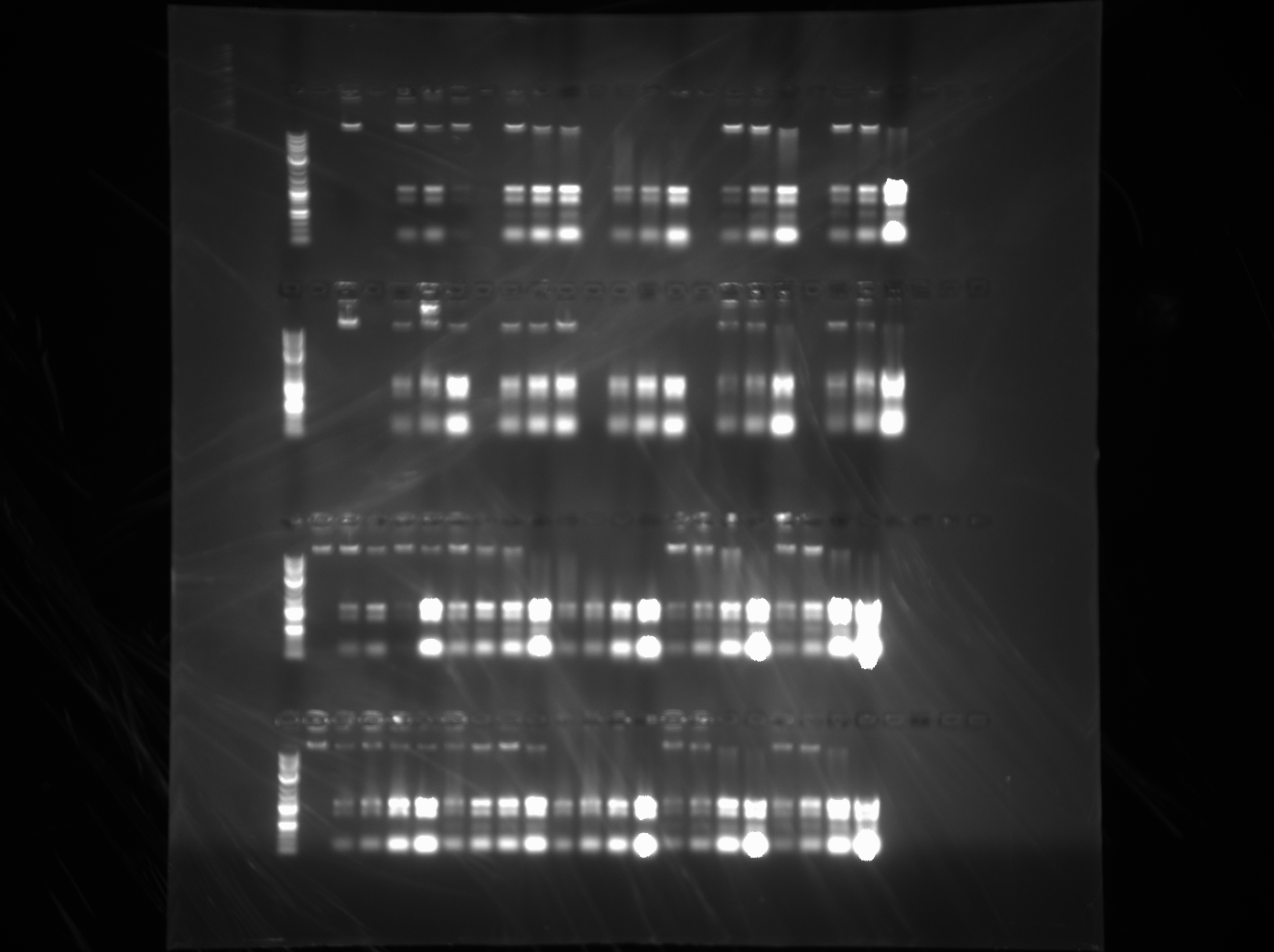

Supplement: Figure 1—source data 2. [file elife-90607-fig1-data2.zip › Figure 1-source data 2/Figure 1F-top and bottom_2018-10-18 18hr 07min.tif]

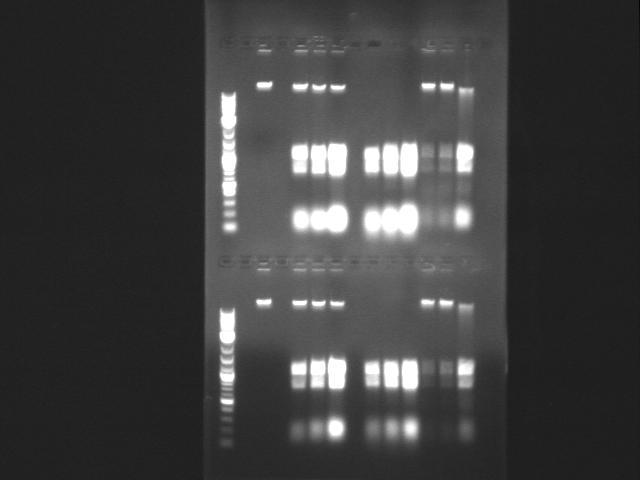

Supplement: Figure 1—source data 2. [file elife-90607-fig1-data2.zip › Figure 1-source data 2/Figure 1E-bottom_2018-12-20 13hr 03min.tif]

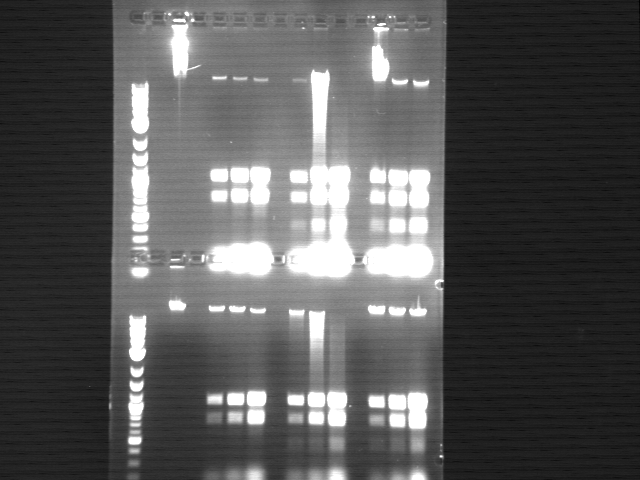

Supplement: Figure 1—source data 2. [file elife-90607-fig1-data2.zip › Figure 1-source data 2/Figure 1B-bottom_2018-09-19 17hr 35min.tif]

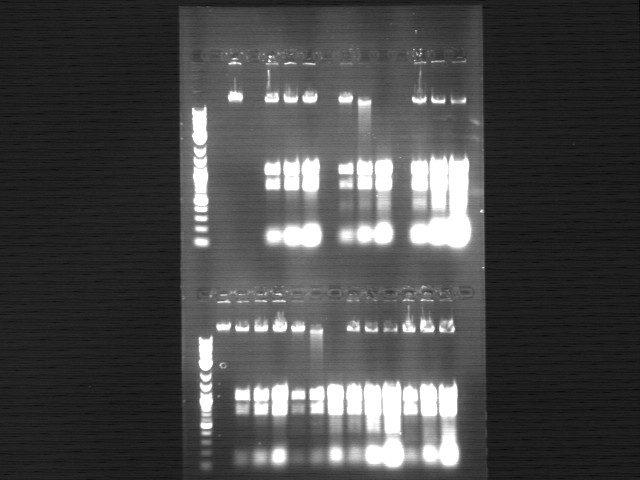

Supplement: Figure 1—source data 2. [file elife-90607-fig1-data2.zip › Figure 1-source data 2/Figure 1B-top_2018-09-07 19hr 37min.tif]

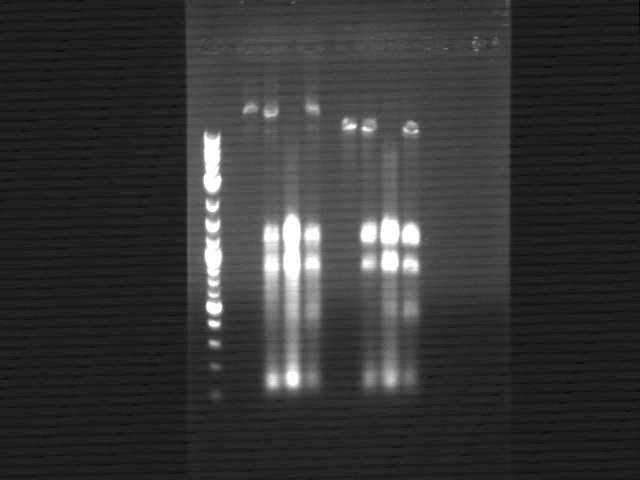

Supplement: Figure 1—figure supplement 1—source data 2. [file elife-90607-fig1-figsupp1-data2.zip › Figure 1-figure supplement 1-source data 2/Figure 1-figure supplement 1D_2018-05-24 21hr 11min.tif]

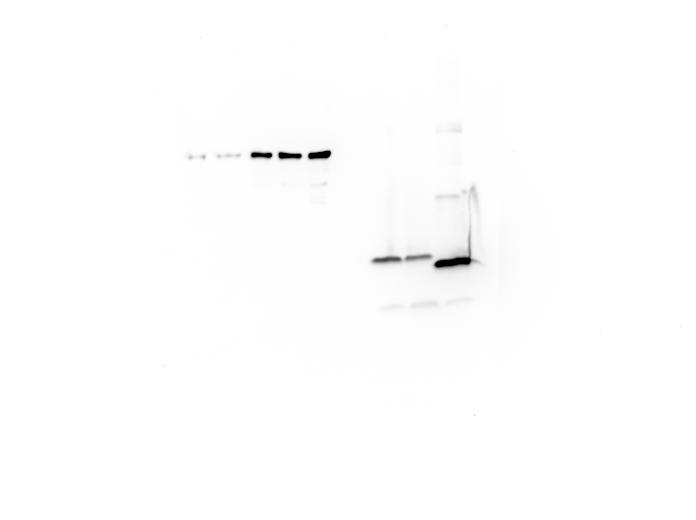

Supplement: Figure 1—figure supplement 1—source data 2. [file elife-90607-fig1-figsupp1-data2.zip › Figure 1-figure supplement 1-source data 2/Figure 1-figure supplement 1C_2018-05-01 18hr 52min v2.tif]

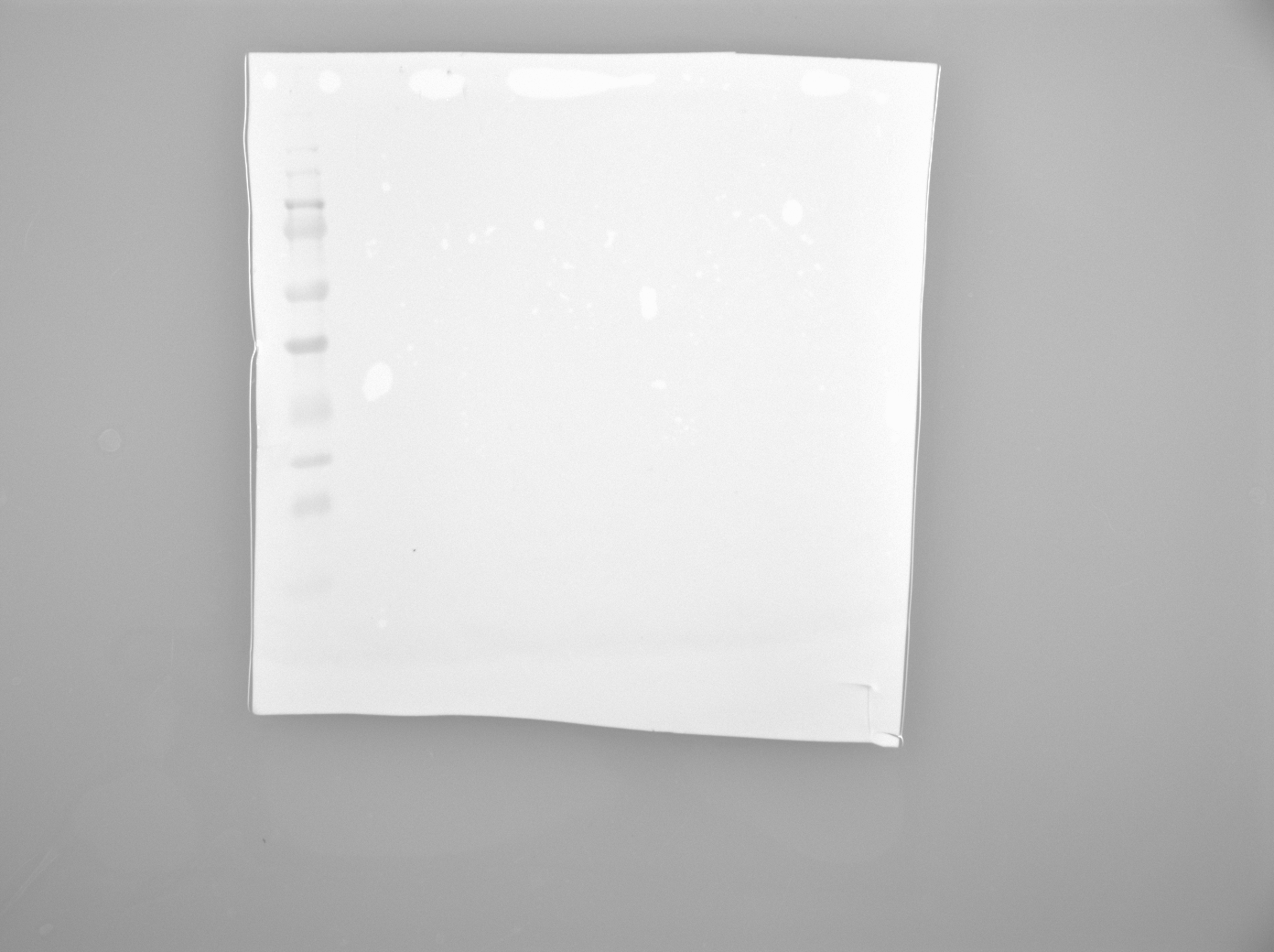

Supplement: Figure 1—figure supplement 1—source data 2. [file elife-90607-fig1-figsupp1-data2.zip › Figure 1-figure supplement 1-source data 2/Figure 1-figure supplement 1C-ladder_2018-05-01 18hr 50min.tif]

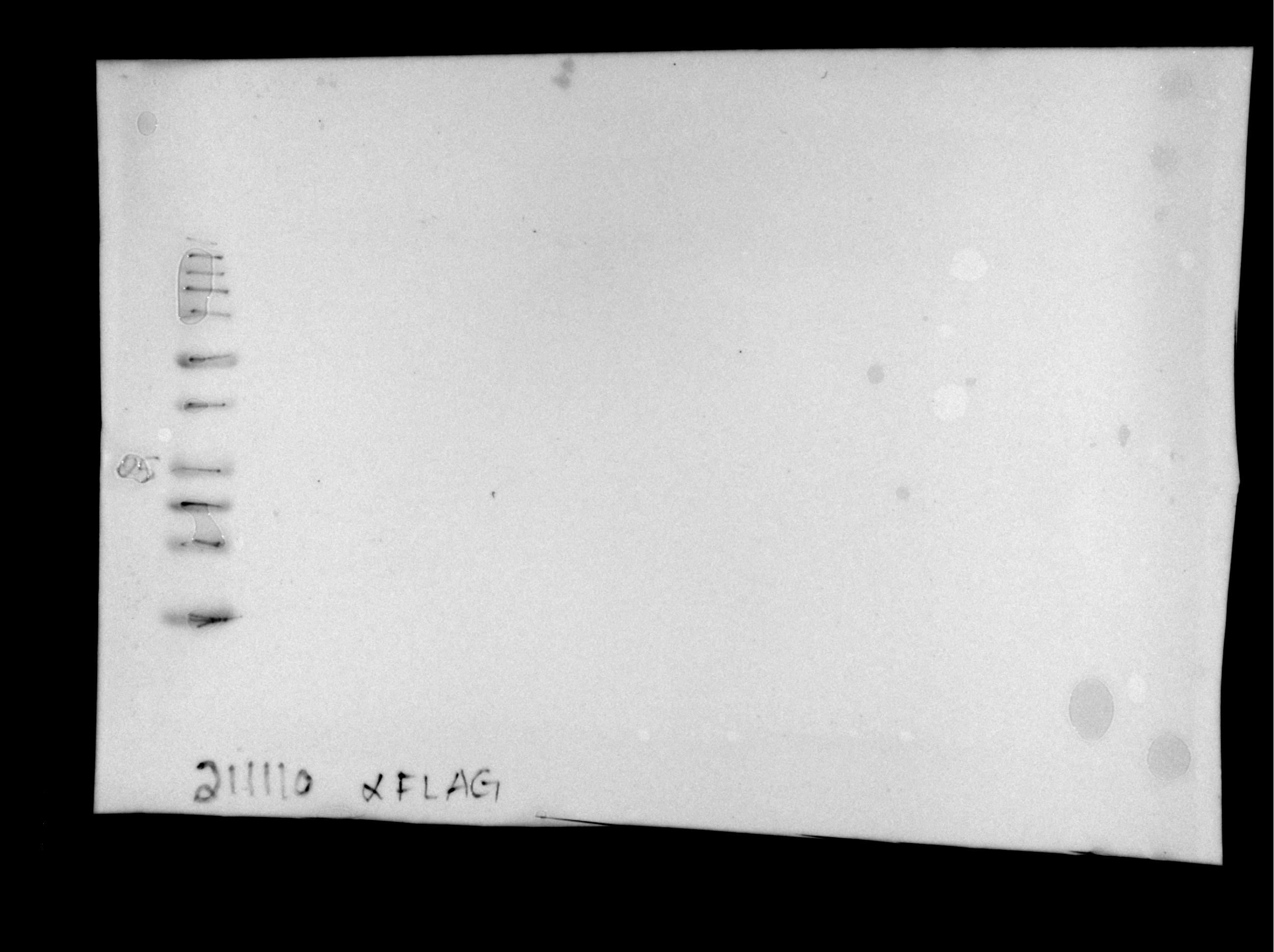

Supplement: Figure 2—source data 2. [file elife-90607-fig2-data2.zip › Figure 2-source data 2/Figure 2E-left gel_211111_anti-FLAG_Colorimetric.tif]

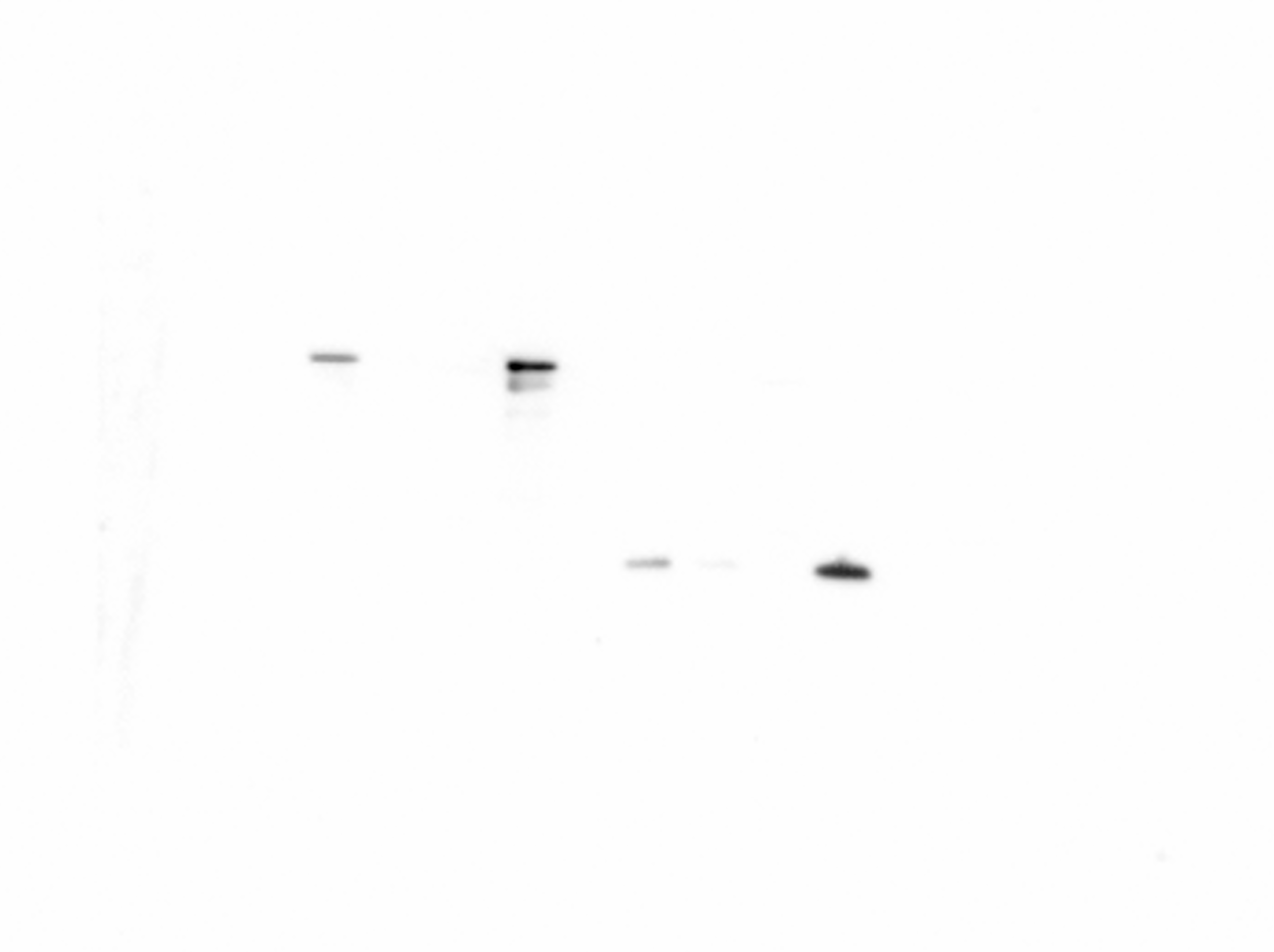

Supplement: Figure 2—source data 2. [file elife-90607-fig2-data2.zip › Figure 2-source data 2/Figure 2E-left gel_211111_anti-FLAG_Chemi.tif]

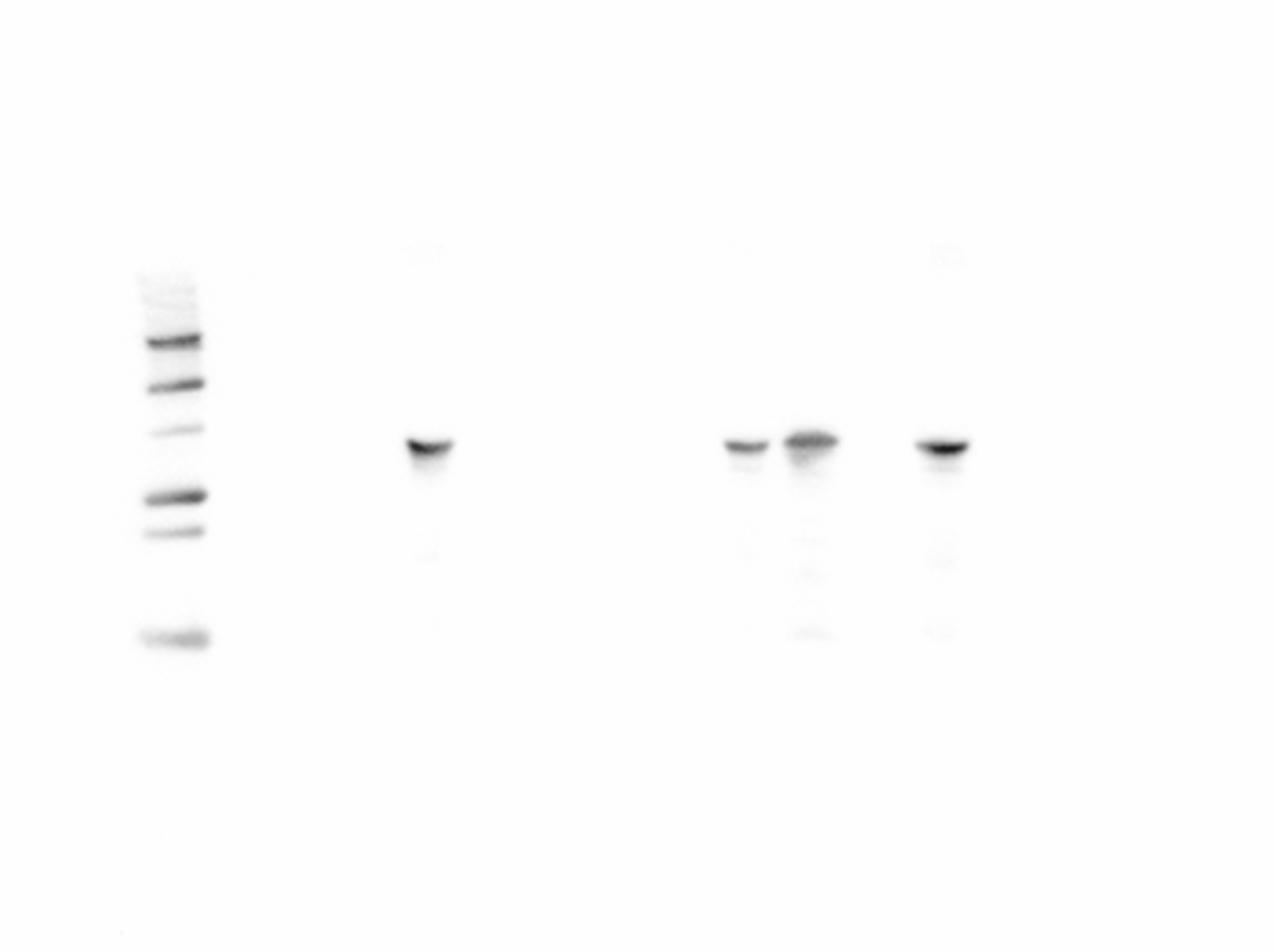

Supplement: Figure 2—source data 2. [file elife-90607-fig2-data2.zip › Figure 2-source data 2/Figure 2E-right gel_211111_anti-StrepII_Chemi.tif]

The orange thick arrows mark the relevant bands.

-- Knecht\*, Sirias\* et al., *eLife*

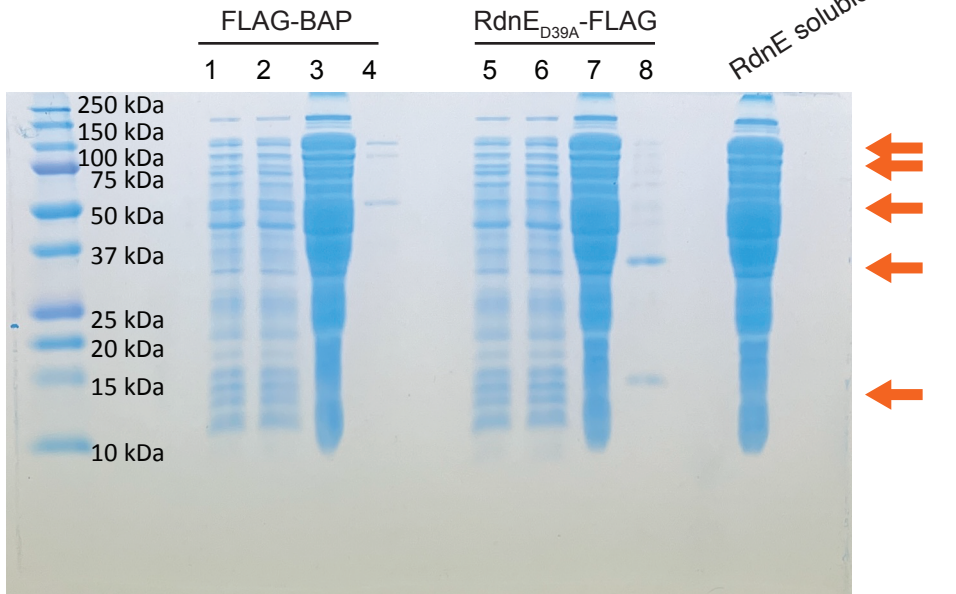

Supplement: Figure 2—figure supplement 1—source data 1. [file elife-90607-fig2-figsupp1-data1.zip › Figure 2-figure supplement 1-source data-source data 1.pdf]

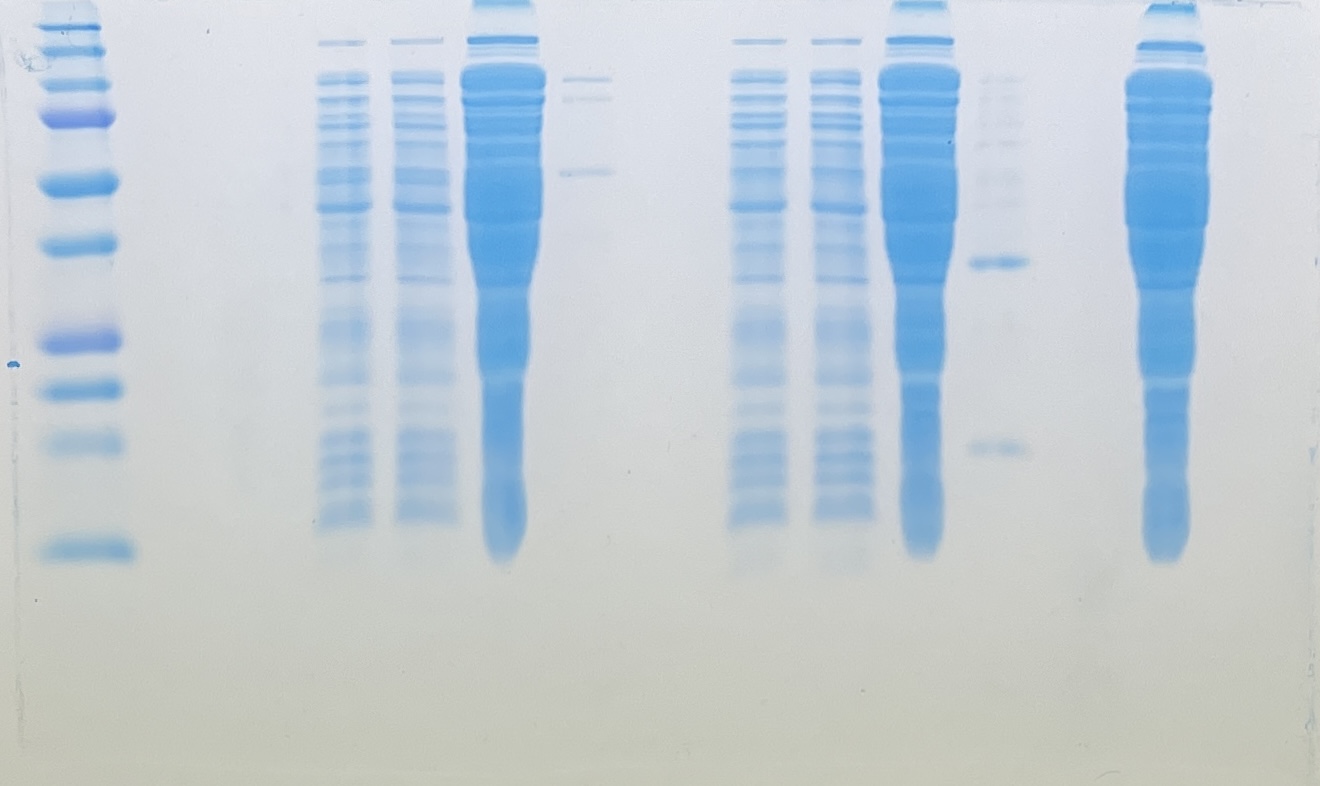

Supplement: Figure 2—figure supplement 1—source data 2. [file elife-90607-fig2-figsupp1-data2.zip › Figure 2-figure supplement 1-source data 2/Figure 2 - Figure supplement 1_211110-coomassie.jpg]

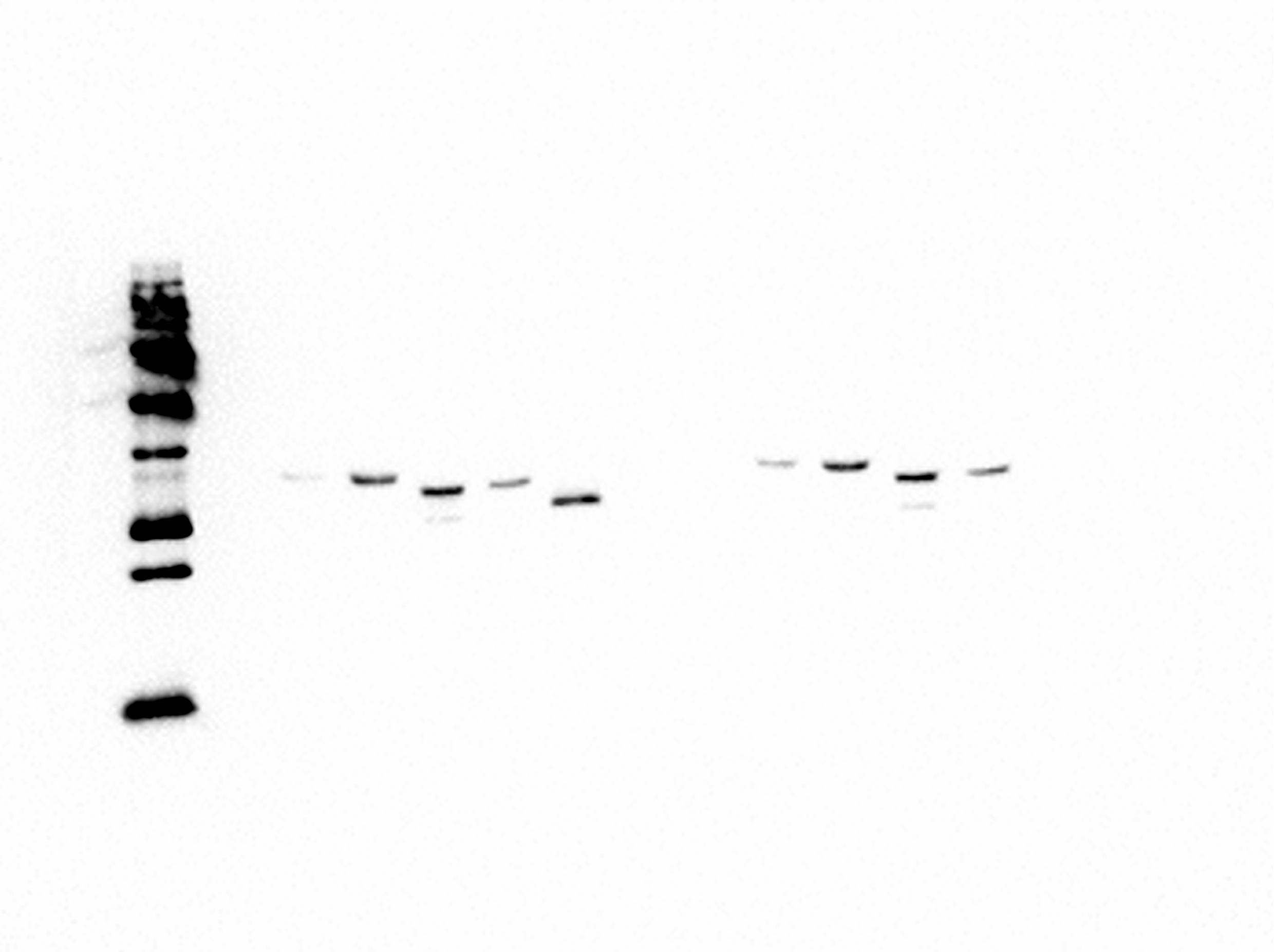

Supplement: Figure 4—figure supplement 2—source data 2. [file elife-90607-fig4-figsupp2-data2.zip › Figure 4-figure supplement 2-source data 2/Figure 4-figure supplement 2-source data_231122_antiST_intensebands_Chemi.tif]

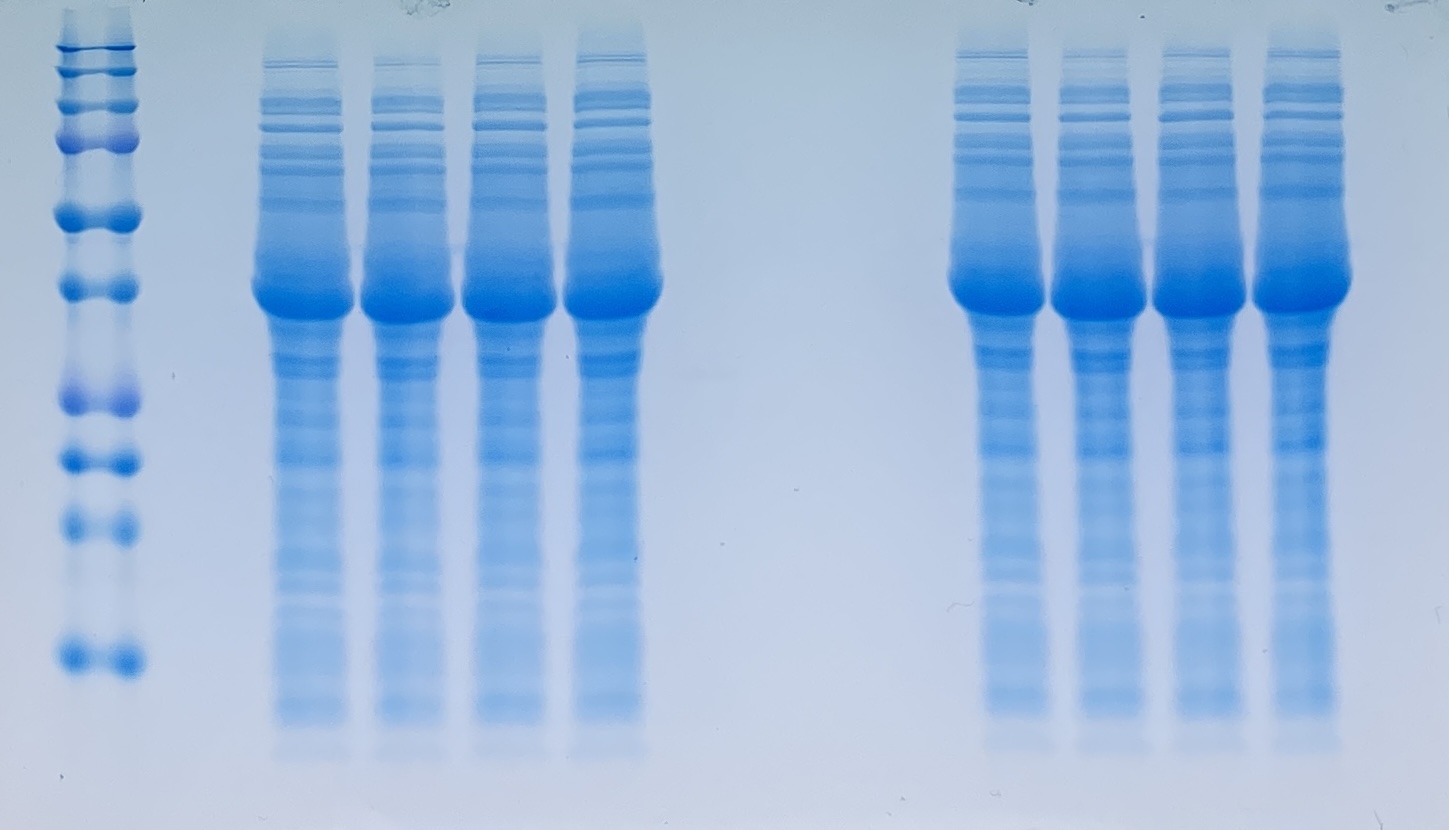

Supplement: Figure 4—figure supplement 2—source data 2. [file elife-90607-fig4-figsupp2-data2.zip › Figure 4-figure supplement 2-source data 2/Figure 4-figure supplement 2-source data_231122-coomassie.jpg]

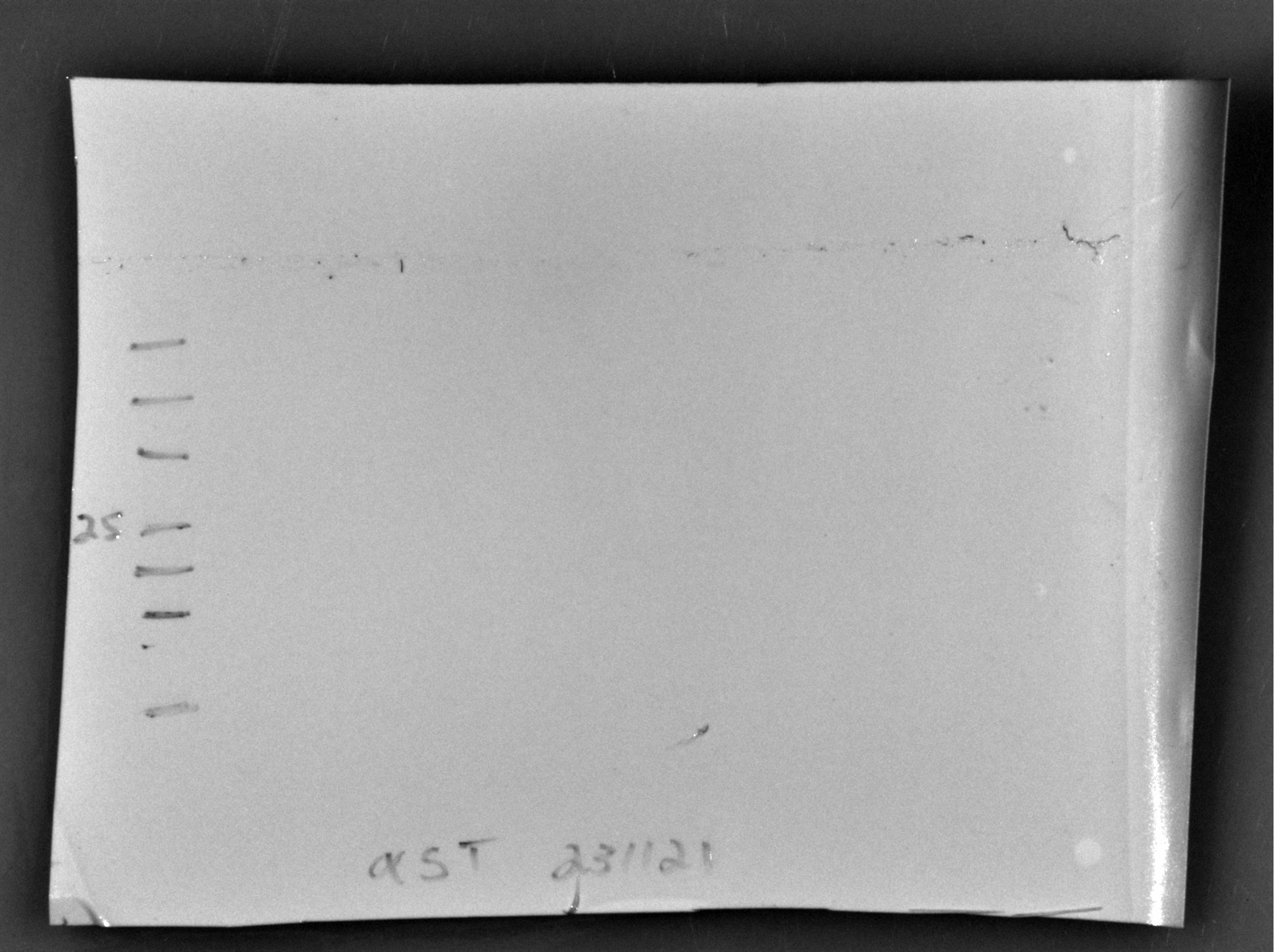

Supplement: Figure 4—figure supplement 2—source data 2. [file elife-90607-fig4-figsupp2-data2.zip › Figure 4-figure supplement 2-source data 2/Figure 4-figure supplement 2-source data_231122_antiST_intensebands_Colorimetric.tif]

-- Knecht\*, Sirias\* et al., *eLife*

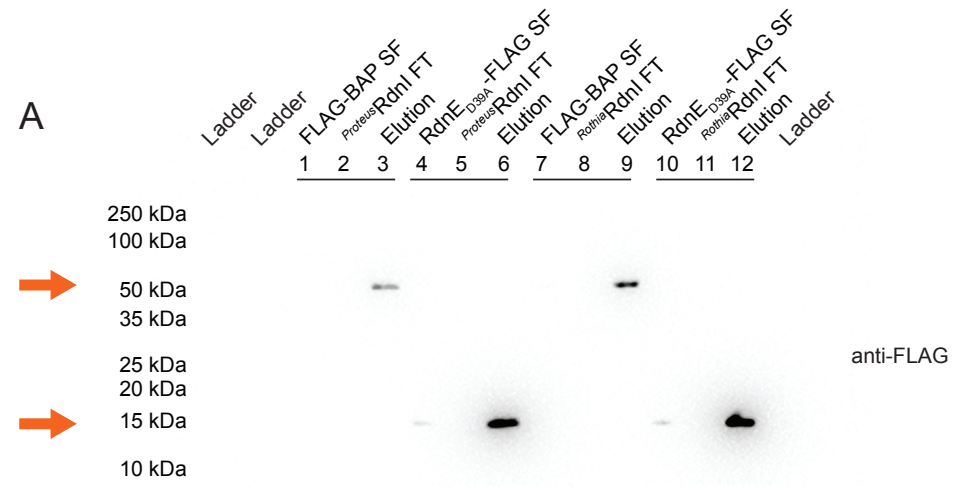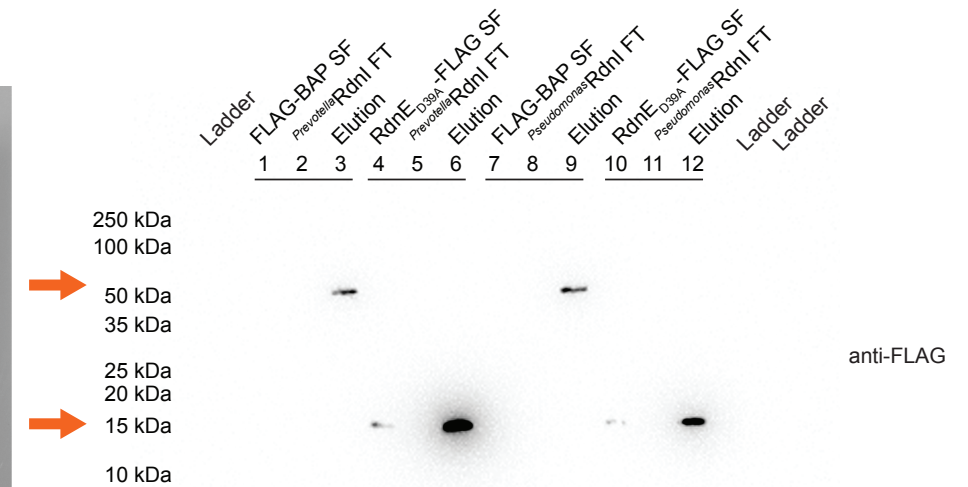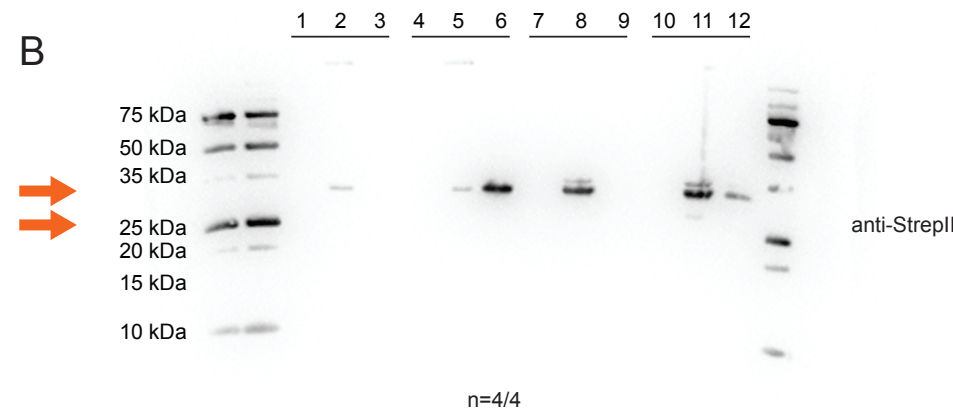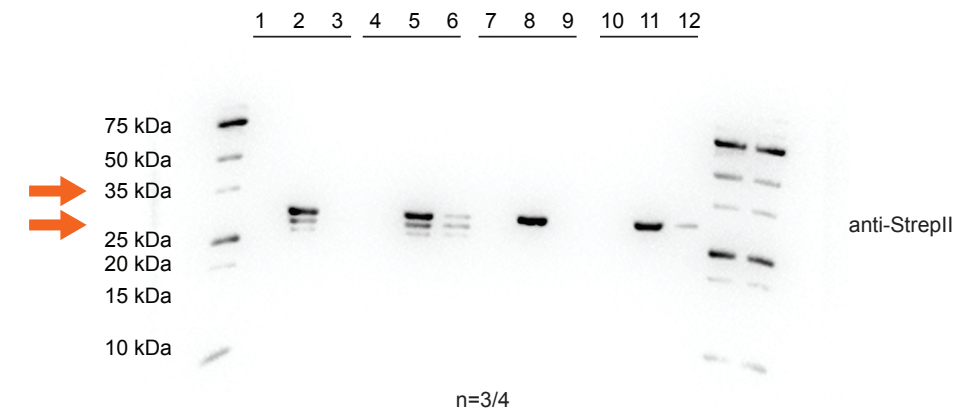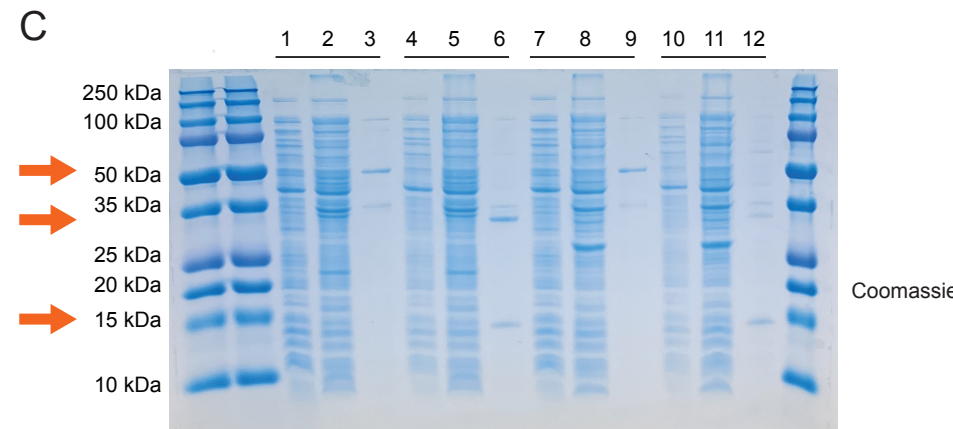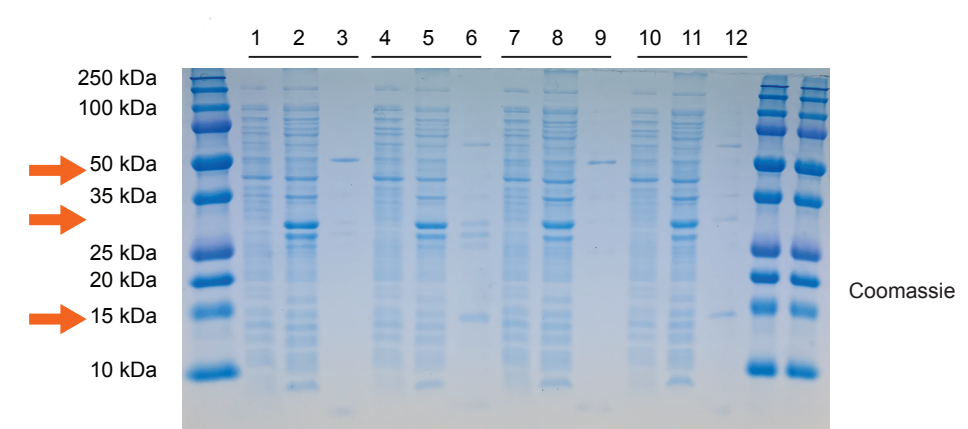

Supplement: Figure 4—figure supplement 3—source data 1. [file elife-90607-fig4-figsupp3-data1.zip › Figure 4-figure supplement 3-source data 1.pdf]

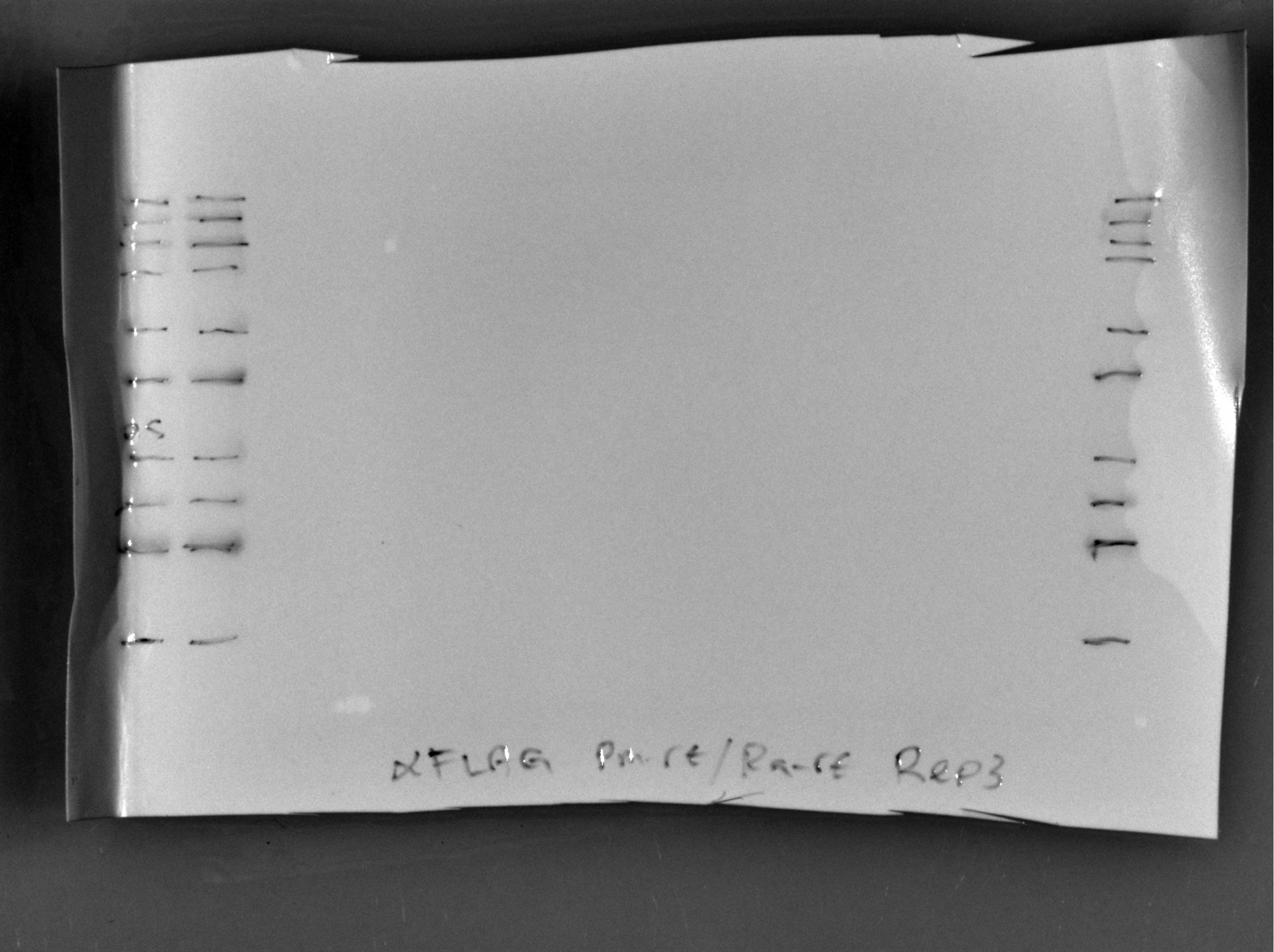

Supplement: Figure 4—figure supplement 3—source data 2. [file elife-90607-fig4-figsupp3-data2.zip › Figure 4-figure supplement 3-source data 2/Figure 4-figure supplement 3-left_220311_anti-FLAG_Colorimetric.tif]

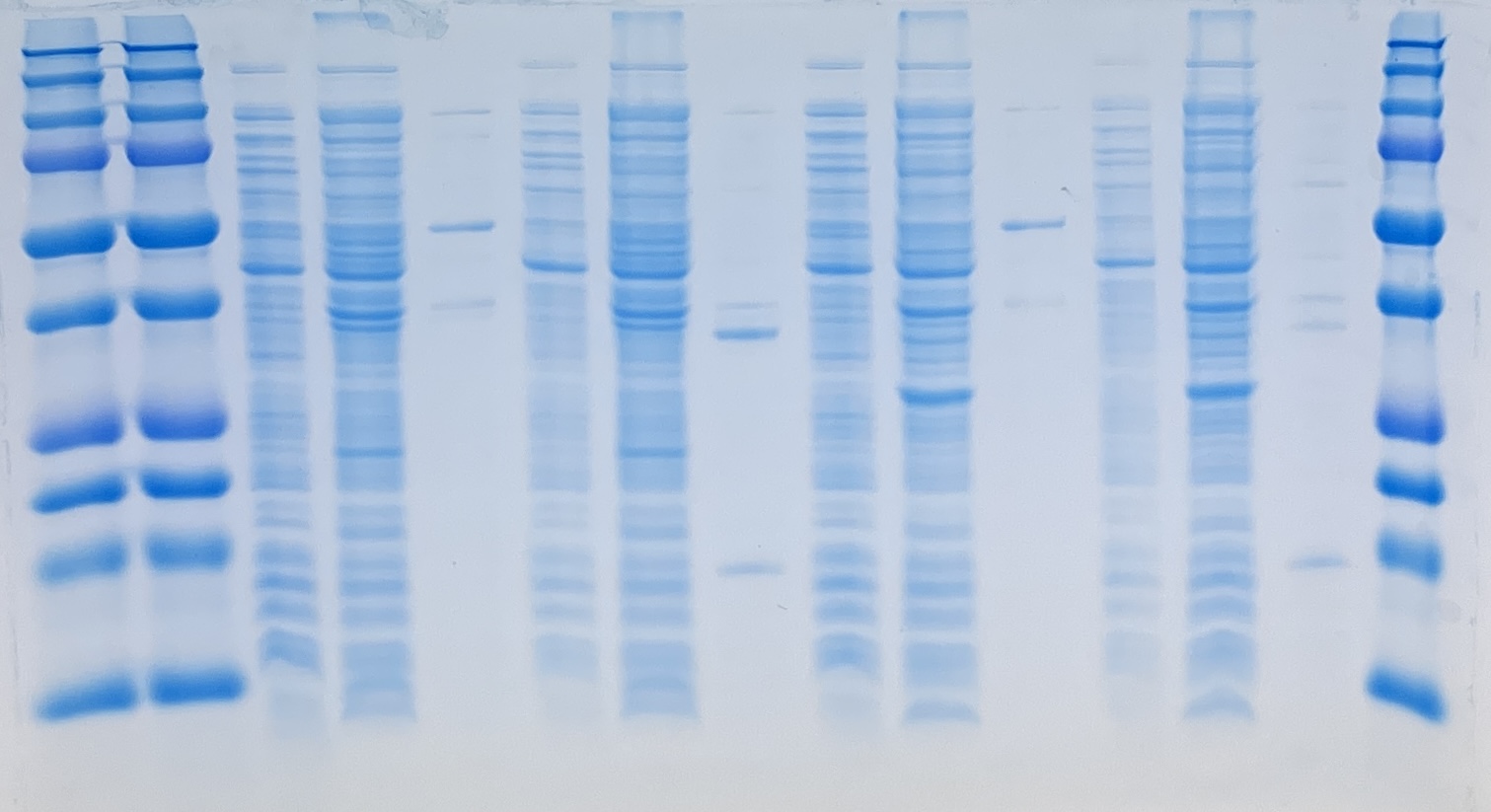

Supplement: Figure 4—figure supplement 3—source data 2. [file elife-90607-fig4-figsupp3-data2.zip › Figure 4-figure supplement 3-source data 2/Figure 4-figure supplement 3-left_220310-coomassie.jpg]

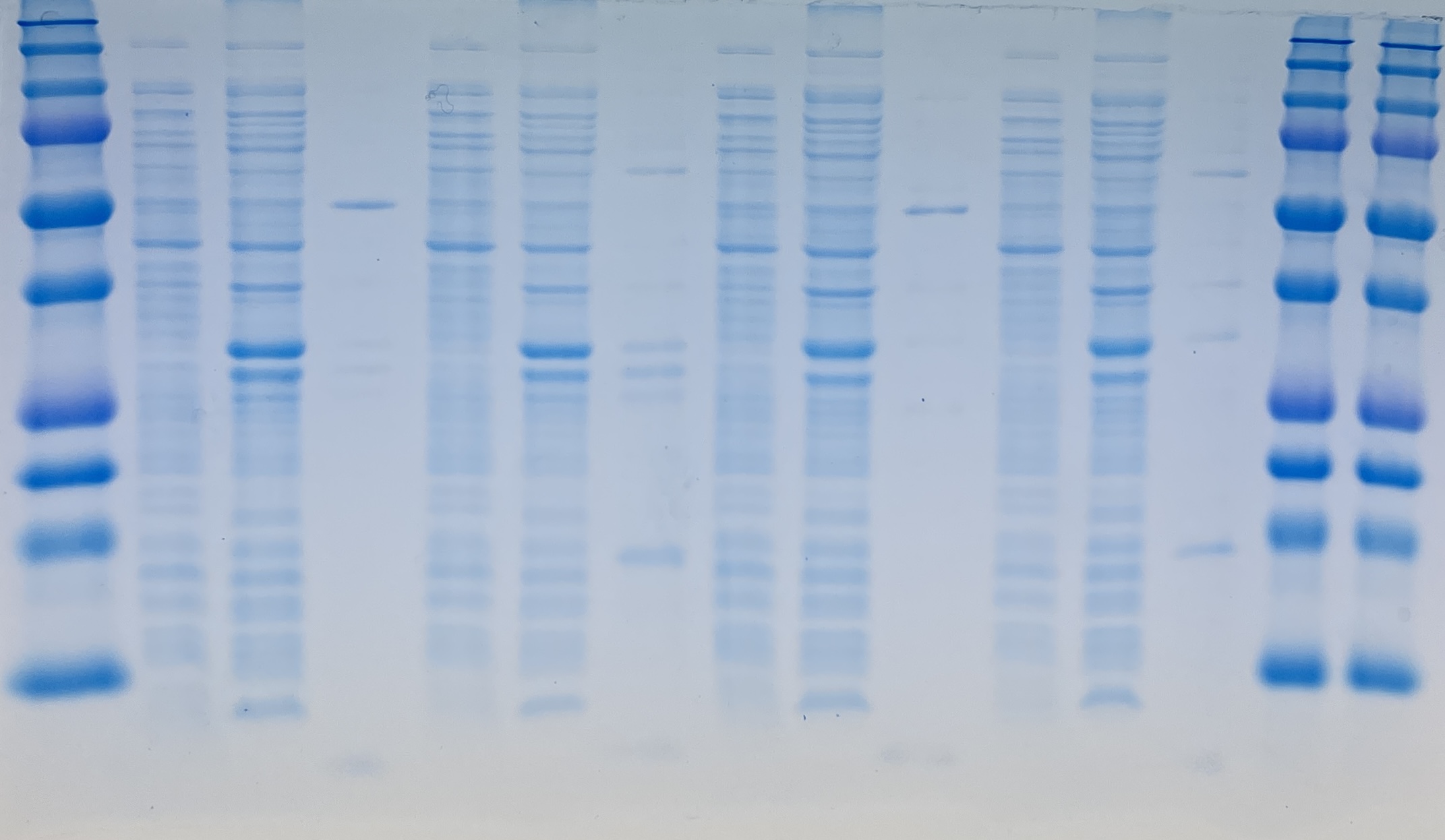

Supplement: Figure 4—figure supplement 3—source data 2. [file elife-90607-fig4-figsupp3-data2.zip › Figure 4-figure supplement 3-source data 2/Figure 4-figure supplement 3-right_220317-coomassie.jpg]

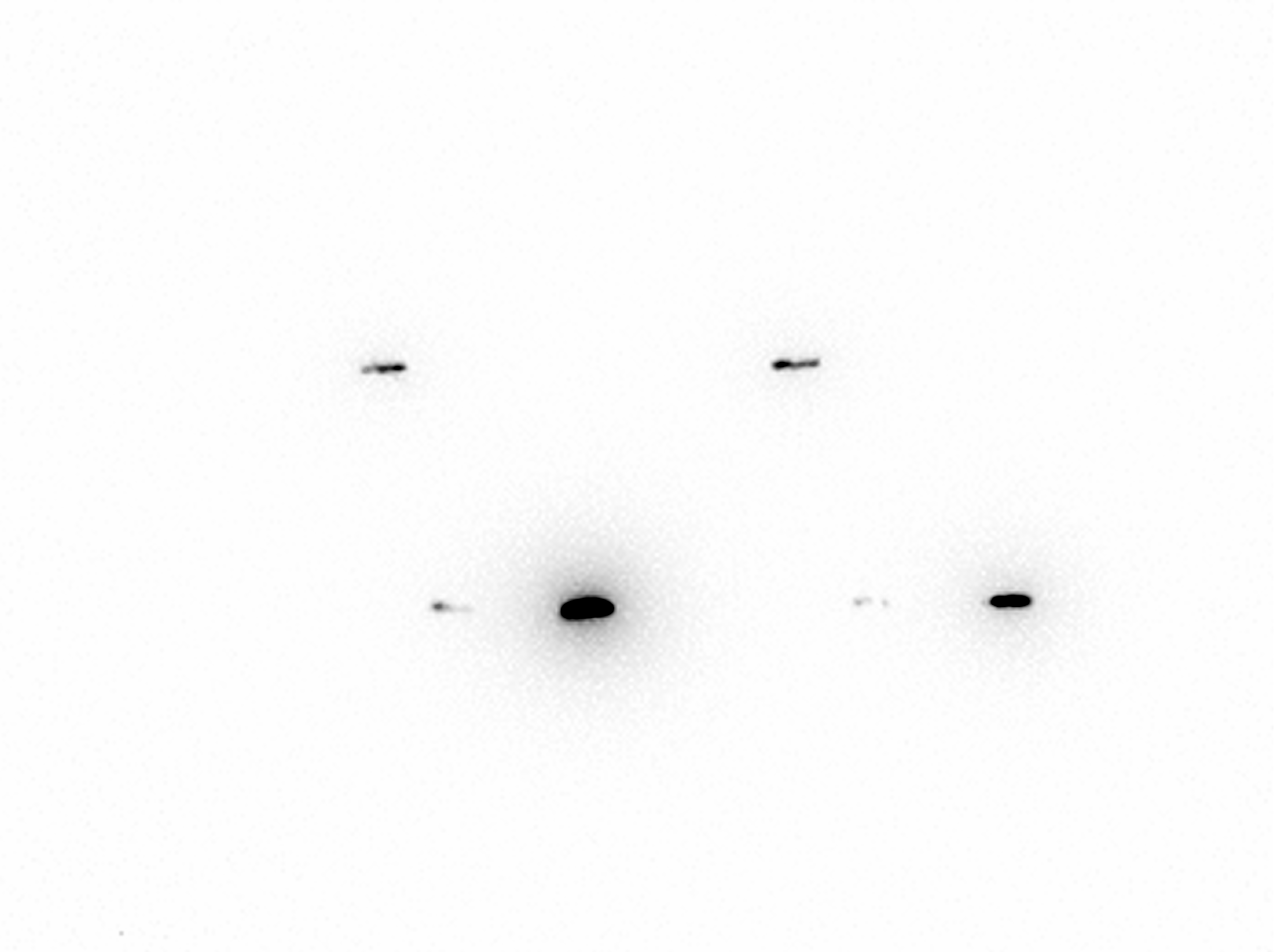

Supplement: Figure 4—figure supplement 3—source data 2. [file elife-90607-fig4-figsupp3-data2.zip › Figure 4-figure supplement 3-source data 2/Figure 4-figure supplement 3-right_2022-03-18-Pj-Pf-bind-rep3-FLAG_Chemi.tif]

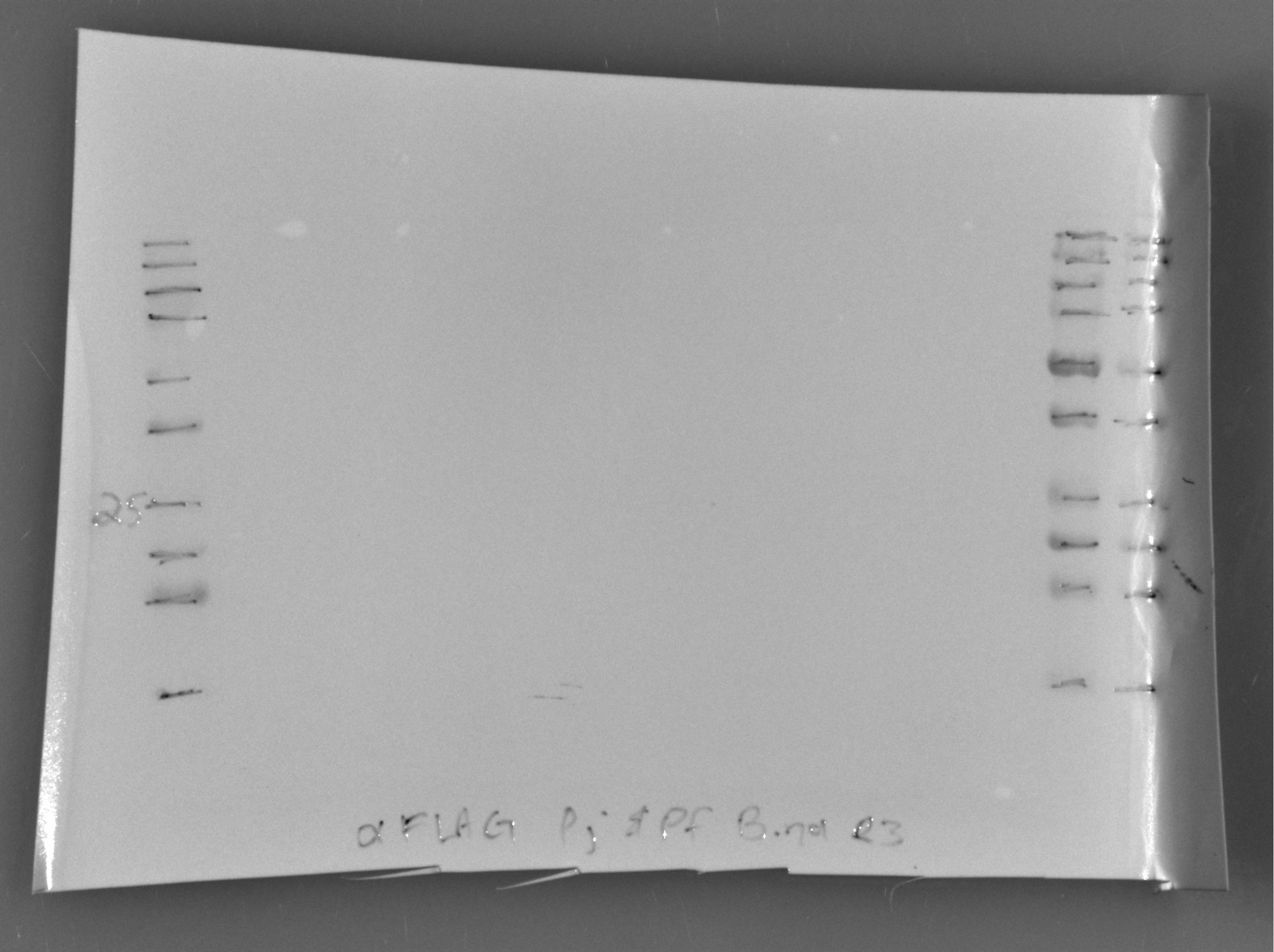

Supplement: Figure 4—figure supplement 3—source data 2. [file elife-90607-fig4-figsupp3-data2.zip › Figure 4-figure supplement 3-source data 2/Figure 4-figure supplement 3-right_2022-03-18-Pj-Pf-bind-rep3-FLAG_Colorimetric.tif]

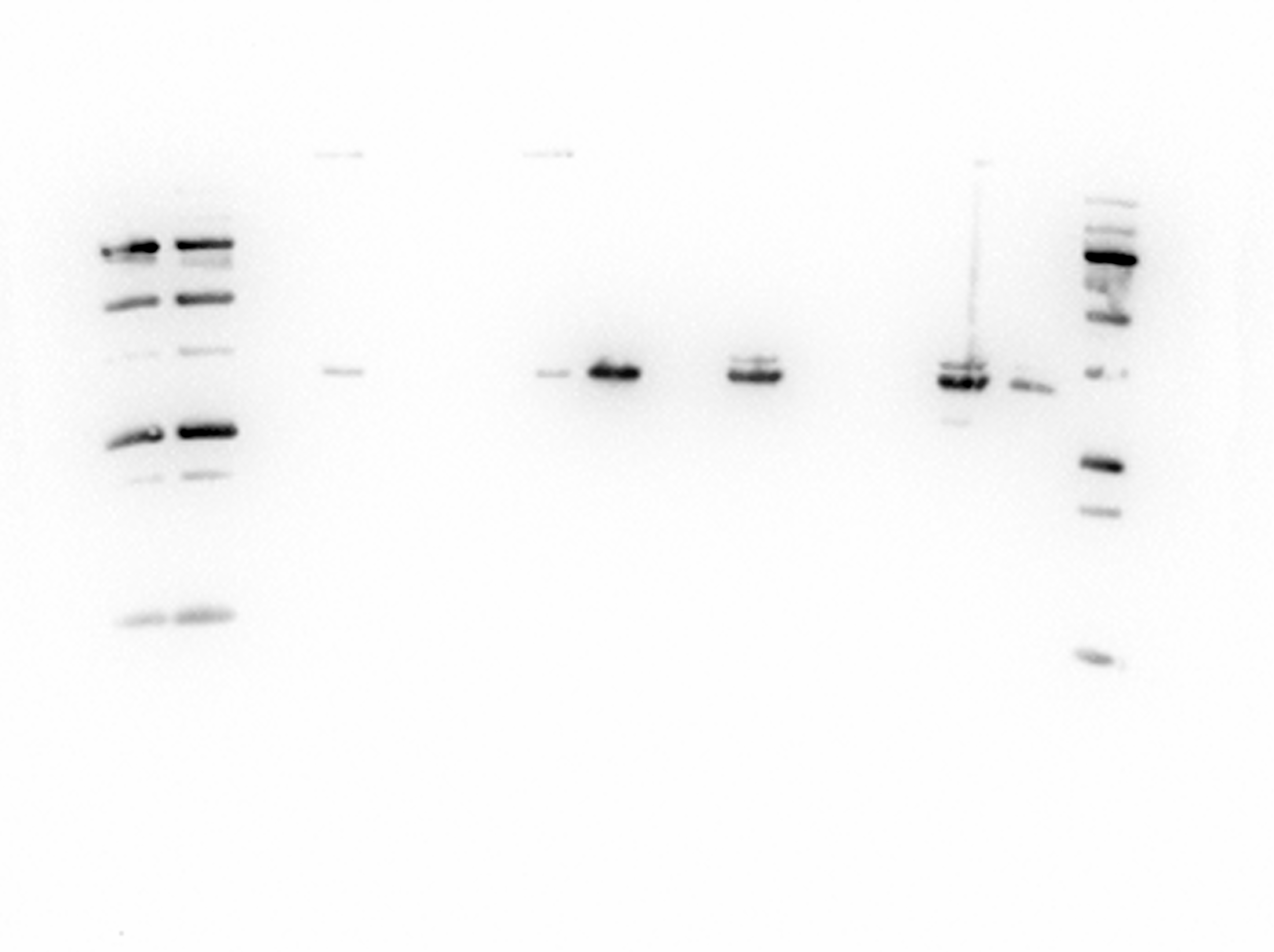

Supplement: Figure 4—figure supplement 3—source data 2. [file elife-90607-fig4-figsupp3-data2.zip › Figure 4-figure supplement 3-source data 2/Figure 4-figure supplement 3-left_220311_anti-StrepII_Chemi.tif]

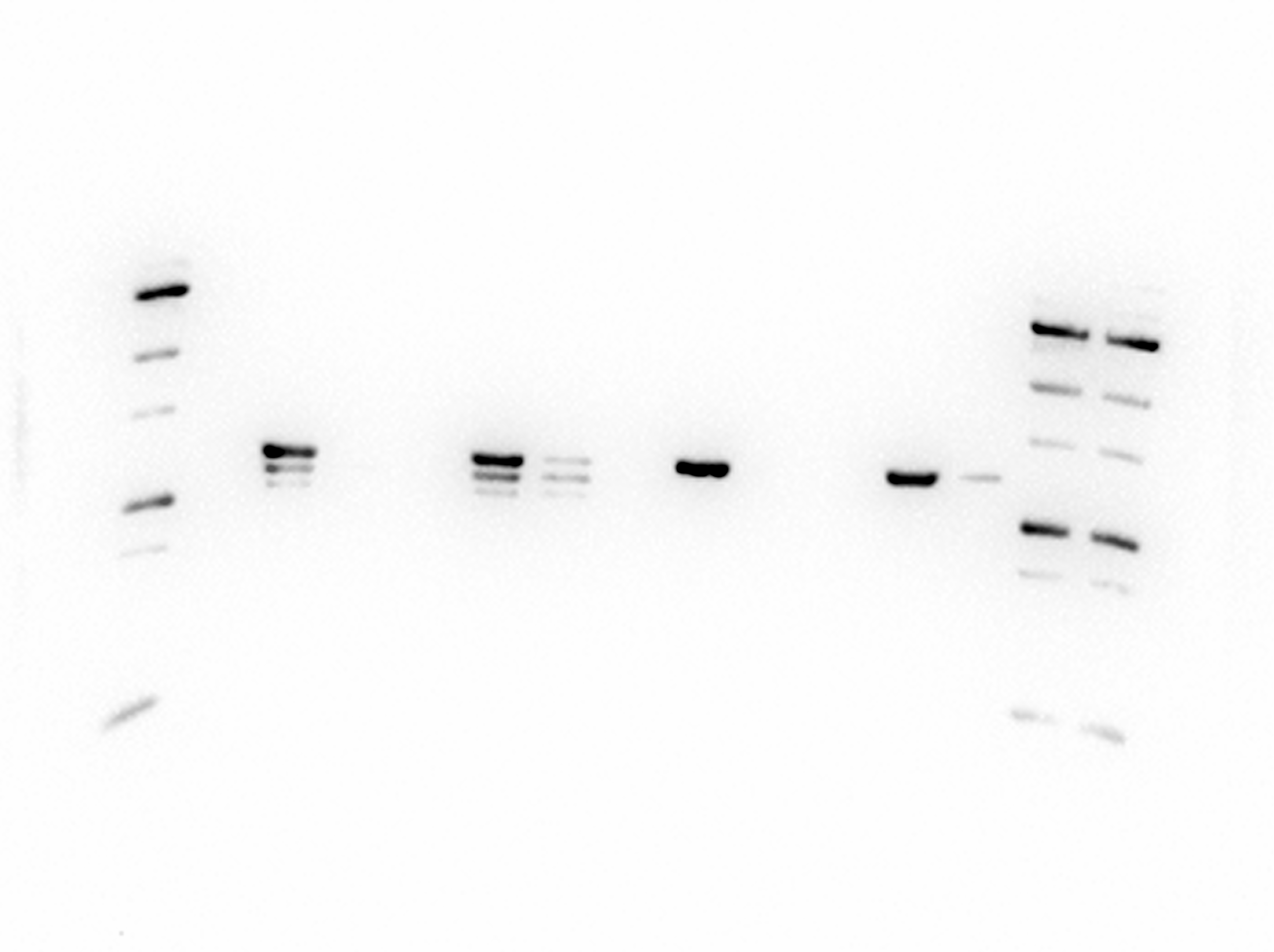

Supplement: Figure 4—figure supplement 3—source data 2. [file elife-90607-fig4-figsupp3-data2.zip › Figure 4-figure supplement 3-source data 2/Figure 4-figure supplement 3-right_2022-03-18-Pj-Pf-bind-rep3-StrepII_Chemi.tif]

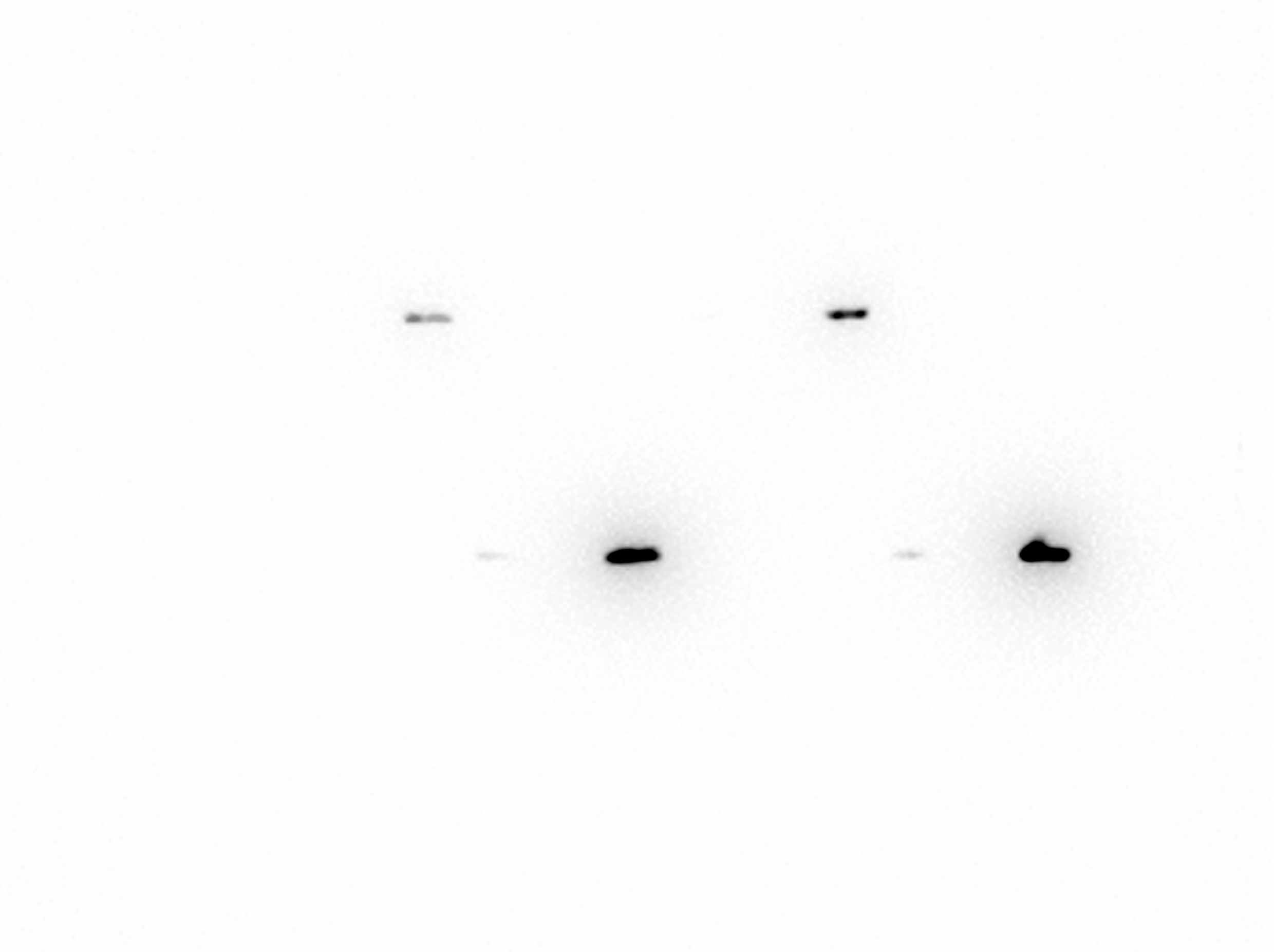

Supplement: Figure 4—figure supplement 3—source data 2. [file elife-90607-fig4-figsupp3-data2.zip › Figure 4-figure supplement 3-source data 2/Figure 4-figure supplement 3-left_220311_anti-FLAG_Chemi.tif]
