# Supplementary material for: Non-cognate immunity proteins provide broader defenses against interbacterial effectors in microbial communities
Source: eLife. 2025 Sep 30;12:RP90607. doi: 10.7554/eLife.90607 (PMC12483513; doi:10.7554/eLife.90607)

The orange thick arrows mark the relevant bands discussed in the text.
-- Knecht*, Sirias* et al., *eLife*

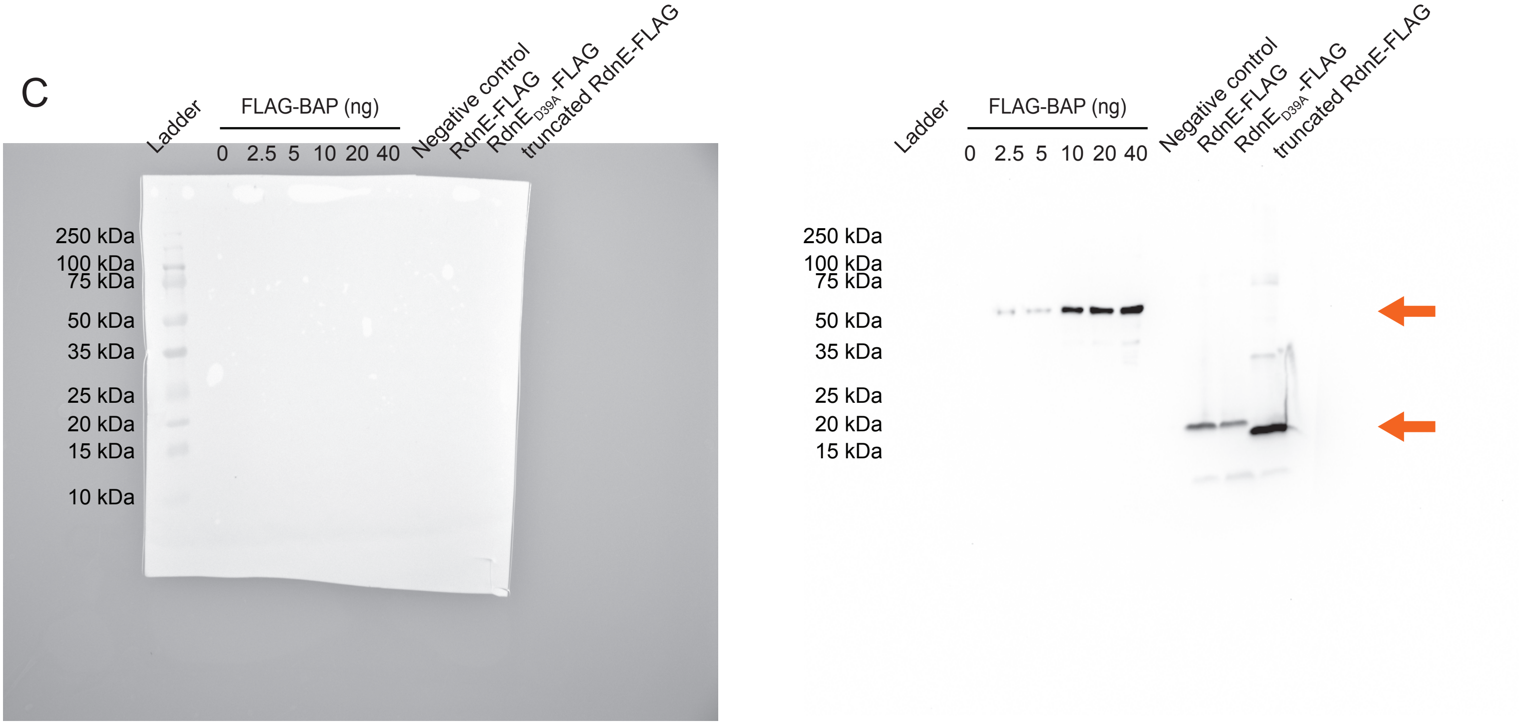

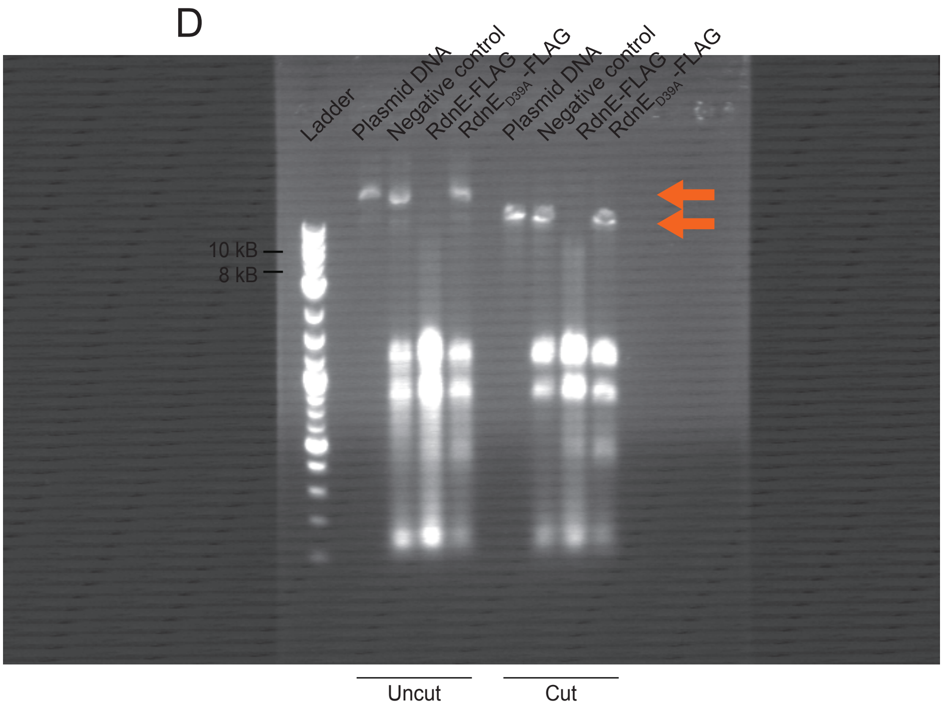

Supplement: Figure 1—figure supplement 1—source data 1. [file elife-90607-fig1-figsupp1-data1.zip › Figure 1-figure supplement 1-source data-source data 1.pdf]
