# Supplementary material for: Non-cognate immunity proteins provide broader defenses against interbacterial effectors in microbial communities
Source: eLife. 2025 Sep 30;12:RP90607. doi: 10.7554/eLife.90607 (PMC12483513; doi:10.7554/eLife.90607)

The orange thick arrows mark the relevant bands discussed in the manuscript.
-- Knecht*, Sirias* et al., *eLife*

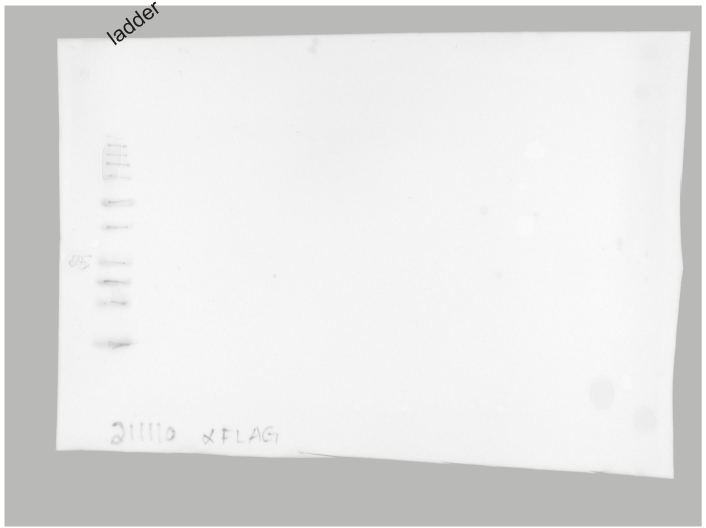

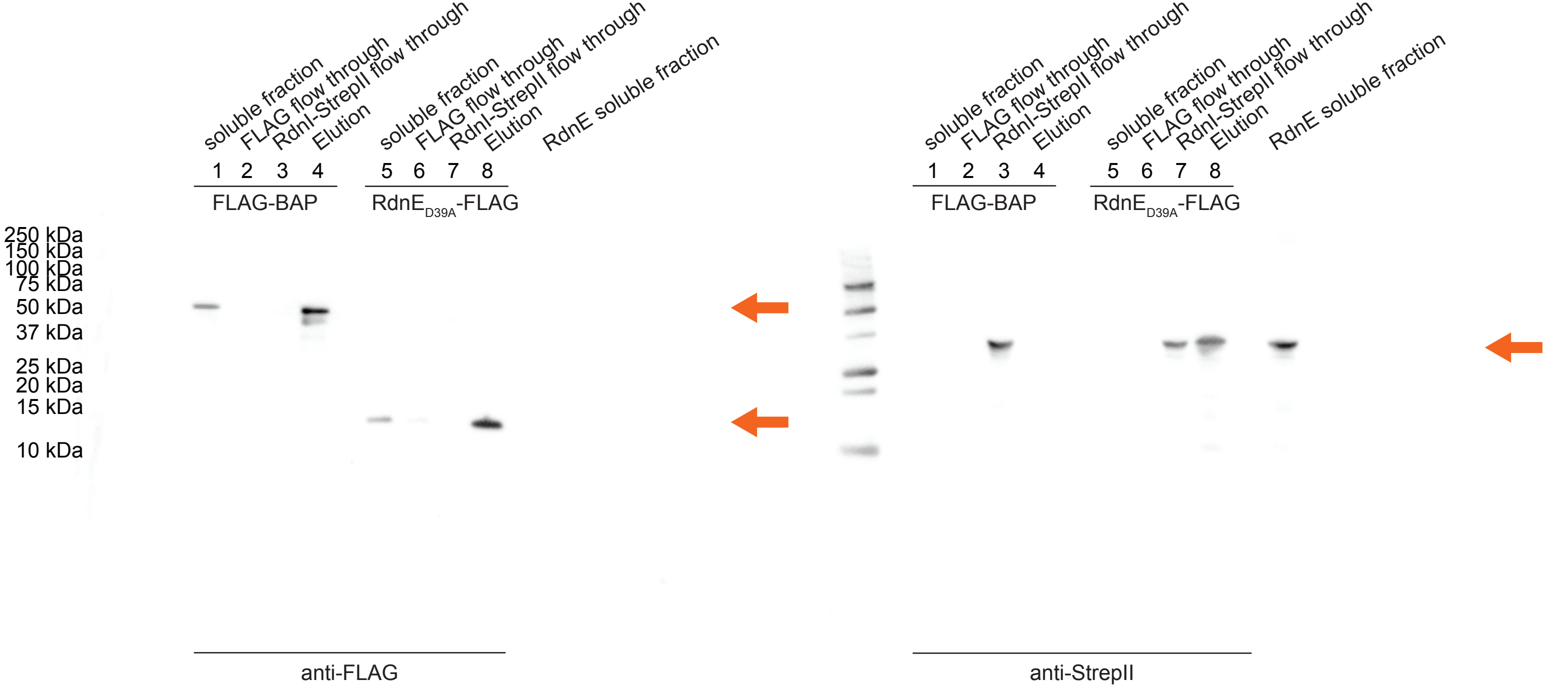

Supplement: Figure 2—source data 1. [file elife-90607-fig2-data1.zip › Figure 2-source data 1.pdf]
