# Supplementary material for: Non-cognate immunity proteins provide broader defenses against interbacterial effectors in microbial communities
Source: eLife. 2025 Sep 30;12:RP90607. doi: 10.7554/eLife.90607 (PMC12483513; doi:10.7554/eLife.90607)

The orange thick arrows mark the relevant bands on the left gel. All visible bands are relevant on the right gel.
-- Knecht*, Sirias* et al., *eLife*

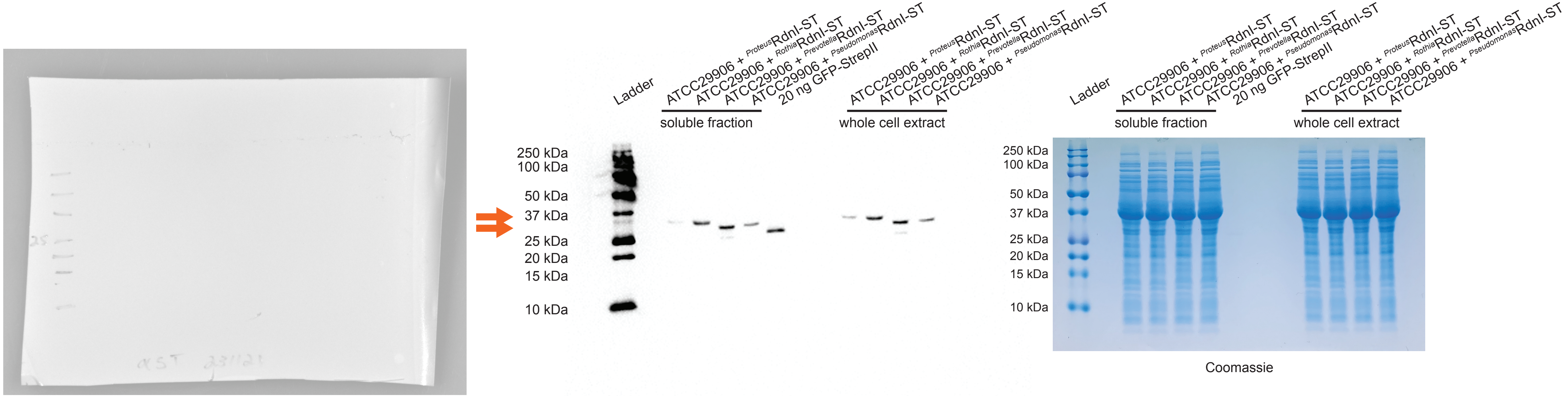

Supplement: Figure 4—figure supplement 2—source data 1. [file elife-90607-fig4-figsupp2-data1.zip › Figure 4-figure supplement 2-source data 1.pdf]
